# Detection of putative new mutacins by bioinformatic analysis using available web tools

**DOI:** 10.1186/1756-0381-4-22

**Published:** 2011-07-14

**Authors:** Guillaume G Nicolas

**Affiliations:** 1 Département de Biochimie Microbiologie et Bioinformatique, Faculté des Sciences et Génie, Université Laval, Québec (Québec), G1K7P4, Canada

## Abstract

In order to characterise new bacteriocins produced by *Streptococcus mutans *we perform a complete bioinformatic analyses by scanning the genome sequence of strains UA159 and NN2025. By searching in the adjacent genomic context of the two-component signal transduction system we predicted the existence of many putative new bacteriocins' maturation pathways and some of them were only exclusive to a group of *Streptococcus*. Computational genomic and proteomic analysis combined to predictive functionnal analysis represent an alternative way for rapid identification of new putative bacteriocins as well as new potential antimicrobial drugs compared to the more traditional methods of drugs discovery using antagonism tests.

## Findings

The increasing resistance of bacteria to antibiotics motives researches for new antimicrobial compounds [1]. In this way bacteriocins which are small antibacterial ribosomally synthetized peptides produced by bacteria represent promising candidates [2,3]. Bacteriocins acted on sensitive cells by punching pores in their membrane. To date, the bacteriocins produced by Gram positive bacteria are grouped in two major classes [4] but four classes are also proposed [5]. Lantibiotic_class I and non-lantibiotic_class II bacteriocins display great diversity with regard to their structures, modes of action, and genetic determinants [4,6]. Typical bacteriocin biosynthesis operons are usually organised as a cluster of genes comprising the prepropeptide coding gene associated with genes for exportation and maturation (ATP-binding cassette (ABC) transporter and sometimes combined to a specific protease), genes conferring immunity to the inhibitory activity to prevent self-killing and occasionally genes involved in regulation of the production of the bacteriocin [6,3]. The expression of the bacteriocin gene cluster is under the control of a two-component signal transduction system (TCS) composed of an histidine kinase (HK) and its associated response regulator (RR) that are usually part of the cluster. The inducer can be either the bacteriocin itself or a bacteriocin-like peptide [7].

Discovery of new bacteriocins traditionally rest upon functionnal assays based on the inhibition of specific target bacteria. Such method is limited and time-consuming regarding the culture condition for bacteriocin production with the indicator strains used. The growing of genomic data makes the detection of new bacteriocin peptides possible by using an *in silico *screening strategy and precise computational analyses. Recently many research teams have bring to light existence of new type of bacteriocins using this strategy [8-11]. Furthermore, a very powerful tool for direct discovery of bacteriocins inside genomic data have been recently develop [12]. However, such tool build on well-known bacteriocins characteristics may overlook detection of new type of bacteriocins as bacteriocins detect by Haft methodoly are not found using BAGEL2 [8,9]. Open reading frame detection and identification coding for short peptides including bacteriocin precursors inside genomes is generally recognised as difficult to perform [13].

Our research group is interested in the discovery of new antibacterial compounds produced by *Streptococcus mutans *and named mutacins [14]. Based on the conserved organisation of bacteriocin biosynthesis operon, we screened the genomic context of the HK/RR genes found in the *S. mutans *UA159 genome to detect new putative bacteriocin-encoding genes (GenBank: AE014133) [15]. Following a profound inspection by bioinformatic analysis using available web tools we were able to identify new putative bacteriocin maturation patchways in the *S. mutans *genome.

**Figure 1:**
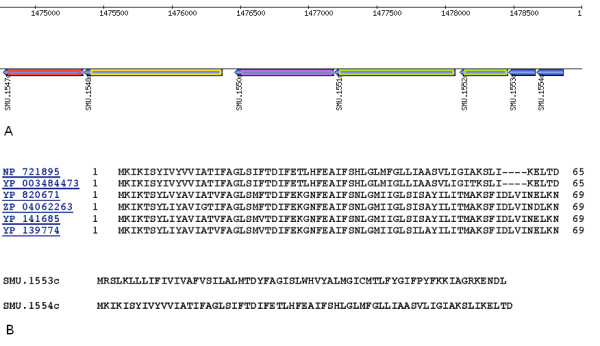
**a) Predicted bacteriocin maturation patchway.** GenBank locus tag are given with the propeptide genes (blue), the ABC transporter genes (green), the immunity gene (majenta), the transcription factor gene (yellow), and the response regulator gene (red). b) Sequence alignment of the significant hits with gi|24379940|ref|NP_721895.1| hypothetical protein SMU.1554c [Streptococcus mutans UA159] as query ID using BlastP and COBALT in default parameters (NCBI).

The Microbial Signal Transduction database (MiST, http://mistdb.com) [16] was used to locate the HK/RR genes inside the *S. mutans *genome (Table 1). A set of small ORF encoding small peptides were identified around each TCS. By browsing the genomic context using the Entrez Gene tool from the NCBI http://www.ncbi.nlm.nih.gov/gene we identified a complete set of bare genes able to produce bacteriocins in the vicinity of the SMU.1548c/1547c locus tag (Figure 1a).

**Table 1:** Two Components Systems found in the *S. mutans *UA159 genome.

HK/RR - Locus tag (NCBI)/gene name	Identifed peptides surrounding the HK/RR	Predicted protein function
SMU.45^#^	SMU.40/41	
SMU.486/487		
SMU.577/576 -	SMU.571	SMU.572
lytS/lytR		dehydrogenase/cyclohydrolase
SMU.660/659		
SMU.928/927		
SMU.1009/1008		
SMU.1037c/1038c	SMU.1047c ?	
SMU.1128/1129 -	SMU.1131c	
ciaH/ciaR		
SMU.1145c/1146c	SMU.1147c	Smu.1148-1150 abc transporter
SMU.1516/1517		
covS/covR		
(vicK/vicR)		
SMU.1548c/1547c	SMU.1553c/1554c	Smu.1550c integrale membrane protein, ...
SMU.1814/1815 -	SMU.1818c	
scnK/scnR		
SMU.1965c/1964c		
SMU.1916/1917 -		
comD/comE		
SMU.1924 - gcrR*		

^# ^HK uncoupled to a RR

* RR uncoupled to an HK

The cluster of genes (location: 1475357-1478860) presents the same genomic organisation than conventional bacteriocin biosynthesis operon with the genes encoding two small peptides (SMU.1554c and SMU.1553c), the ABC transporter genes (SMU.1552c/SMU.1551c), a gene encoding an integrale membrane protein possibly involved in the immunity function (SMU.1550c), and the TCS genes, HK gene (SMU.1548c) and RR gene (SMU.1547c), probably implicated in the regulation of the biosynthesis of the bacteriocin. Furthermore additionnal untypical genes were identified: a methionine aminopeptidase (ampM/SMU.1556c) and a putative acetyltransferase (SMU.1558c) with related function to proteases and scaffoldingproteins.

Putative precursor peptides were analysed for the presence of a signal peptide using Signal-3L http://www.csbio.sjtu.edu.cn/bioinf/Signal-3L/[17] and PrediSi http://www.predisi.de/[18] algorithms.

The potential of antimicrobial activity of the putative mature peptides was evaluated using freely web available programs such as APD2 http://aps.unmc.edu/AP/main.php[19] and the AntiBP2 server http://www.imtech.res.in/raghava/antibp/[20]. Similarity with known antimicrobial peptides was retrieved for the query input peptide sequences. SMU.1553c presents similarity with the carnocyclin A peptide [21].

A BlastP analysis [22] of the precursor peptides reveals the strict conservation of these peptides with their genomic context to the *Streptococcus salivarius *group species (Figure 1b).

**Figure 2:**
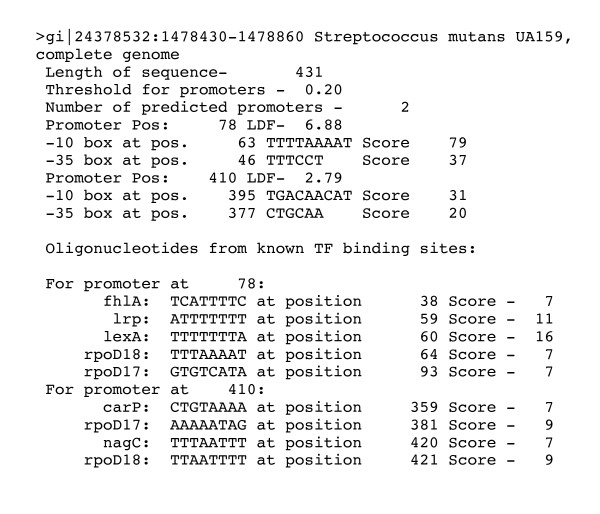
**Report of the BPROM promoter detection software**.

Upstream genomic coding sequence was analyse to detect putative promoter regions and transcription factor binding sites using the bacterial promoter recognition program BPROM (Softberry inc.) (Figure 2).

Many putative mutacin-encoding genes have been previously predicted using bioinformatic analyses and some of them were functionnally verified using mutational analyses for *S. mutans *UA159 [23]. Inactivation of all putative mutacin genes did not abrogate complete antibacterial activity of the strain, let suggest the existence of an other type of inhibitory substance produced which confort the reliability of our hypothesis and findings hither [23].

The group of genes detect by our method predicted the existence of a putative bacteriocin maturation pathway in an exclusive group of *Streptococcus *and reveals its potential to encode for a new type of bacteriocin. It also provides mature hypothesis that may be test by a focused wet lab experiment. Since inactivation of small genes remains difficult to perform, our method study provides a computational evidence for identification of a new putative bacteriocin production. This method can be applied to a large set of short coding sequence with unknown function yet found in the streptococcal genomes [13].

## Abbreviations

ABC: ATP-binding cassette; BlastP: Basic Local Alignment Search Tool for protein; ORF: Open Reading frame; TCS: Two-component signal transduction system; HK: Histidine Kinase; RR: response regulator.

## Competing interests

The author declares that they have no competing interests.

## Authors' contributions

GGN performed the bioinformatic analyses, interpreted the results, and wrote the manuscript.
